# Determinants of COVID-19 vaccine uptake among healthcare workers and the general population in Cyprus

**DOI:** 10.1093/eurpub/ckac131.060

**Published:** 2022-10-25

**Authors:** K Giannakou, G Fakonti, M Kyprianidou

**Affiliations:** Department of Health Sciences, European University Cyprus, Nicosia, Cyprus

## Abstract

**Background:**

Vaccination is a critical intervention in the fight against the coronavirus disease 2019 (COVID-19) pandemic. Various levels of COVID-19 vaccination acceptance have been observed around the world. However, a high percentage of the general population and healthcare professionals (HCPs), refuse the COVID-19 vaccination. This study aims to examine the factors influencing COVID-19 vaccine uptake among HCPs and the general population in Cyprus.

**Methods:**

An online cross-sectional study was conducted, using a self-administered questionnaire to collect information covering various potential determinants including sociodemographic and health-related characteristics, trust in the healthcare system, satisfaction with it, utilization of preventive healthcare services, COVID-19 vaccination information, and general vaccination knowledge.

**Results:**

A total of 2582 participants completed the survey, with 53.5% of individuals in the general population, and 70.0% of the HCPs received the COVID-19 vaccination. We found that as the age increases by one year among the general population, the odds of being vaccinated against COVID-19 increase by 1.02 units (95% 1.00-1.03, p-value=0.035), whilst those with increased trust in national healthcare authorities’ guidelines (OR = 3.96, 95% CI: 3.41-4.61) and increased vaccination knowledge scores (OR = 1.11, 95% CI: 1.05-1.18) were significantly more likely to be vaccinated. Furthermore, male HCPs (OR = 1.91, 95% CI: 1.01-3.59), and those who reported increased trust in national healthcare authorities’ guidelines (OR = 5.38, 95% CI: 3.65-7.95) were significantly more likely to be vaccinated.

**Conclusions:**

Public health policymakers can use national campaigns and long-term planning to build public trust in national healthcare authorities and educate and raise awareness about the benefits of vaccination. Such strategies could pave the way for adequate vaccine uptake and prepare the public for unfavorable scenarios, such as future pandemics.

**Key messages:**

• Our results revealed the importance of vaccination knowledge and trust in healthcare system in respect to COVID-19 vaccination uptake.

• The urgent need for national campaigns and long-term planning to build public trust in national healthcare authorities.

